# Canonical and Alternative Pathways (Insulin and Exercise) of GLUT4 Synthesis, Signaling, Intracellular Clustering, and Recruitment to the Plasma Membrane

**DOI:** 10.3390/ijms27083475

**Published:** 2026-04-13

**Authors:** Arnulfo Ramos-Jiménez, Mariazel Rubio-Valles, Jaime Guereca-Arvizuo, Marco A. Juárez-Oropeza, Javier A. Ramos-Hernández, Isaac A. Chávez-Guevara, Everardo González-Rodríguez, Verónica Moreno-Brito, Rosa P. Hernández Torres

**Affiliations:** 1 Institute of Biomedical Sciences, Autonomous University of Ciudad Juarez, Ciudad Juarez Campus, Chihuahua 32310, Mexico; jaime.guereca@uacj.mx; 2 Faculty of Physical Culture Sciences, Autonomous University of Chihuahua, University Circuit, Campus II, Chihuahua 31125, Mexico; rhernant@uach.mx; 3 Department of Biochemistry, School of Medicine, National Autonomous University of Mexico, Mexico City 04510, Mexico; majo_ya@yahoo.com.mx; 4 Faculty of Medicine, Autonomous University of Nuevo Leon, Monterrey 64460, Mexico; javier.ramoshrn@uanl.edu.mx; 5 Faculty of Sports Ensenada, Autonomous University of Baja California, Ensenada 22860, Mexico; isaac.chavez.guevara@uabc.edu.mx; 6 Faculty of Medicine and Biomedical Sciences, Autonomous University of Chihuahua, University Circuit, Campus II, Chihuahua 31109, Mexico; evegonzal@uach.mx (E.G.-R.); vmoreno@uach.mx (V.M.-B.)

**Keywords:** GLUT4, SLC2A4, vesicular translocation, insulin signaling, PI3K/Akt, AMPK, exercise, muscle contraction, CaMKII, p38 MAPK, insulin resistance, Type 2 diabetes

## Abstract

Glucose transporter type 4 (GLUT4), encoded by the *SLC2A4* gene, is the final effector of insulin-stimulated glucose uptake in insulin-sensitive tissues: skeletal muscle, adipose tissue, and cardiac muscle. Its dynamic localization, retained intracellularly under basal conditions and extensively translocated to the plasma membrane upon stimulation, makes it a master regulator of glycemic homeostasis. While the canonical insulin pathway (PI3K/Akt/TBC1D4) is the most potent and specific mechanism in the postprandial state, its dysfunction is centrally associated with insulin resistance and type 2 diabetes mellitus (T2DM). Crucially, robust alternative signaling networks function completely independently of insulin to regulate GLUT4 synthesis and translocation. Prominent among these are contraction-mediated pathways in skeletal muscle, which employ calcium signaling (via CaMKII), mechanical/metabolic stress sensors (via p38 MAPK γ/δ), and AMP-activated protein kinase (AMPK). This review critically integrates current knowledge, linking the molecular architecture and post-translational modifications of GLUT4 to the complex, tissue-specific signaling networks that govern its vesicular trafficking. We emphasize the hierarchy, redundancy, and interdependence of these pathways, highlighting differences between acute translocation and chronic transcriptional adaptations. Finally, we discuss how deciphering insulin-independent mechanisms offers promising therapeutic opportunities, particularly in identifying pharmacological targets that mimic the metabolic benefits of physical exercise.

## 1. Introduction

Glucose is the main energy source of mammalian cells. The American Diabetes Association states that in healthy individuals, fasting blood glucose levels should stay below 100 mg/dL (range: 70–99 mg/dL) [1]. Glucose enters insulin-sensitive cells through the glucose transporter type 4 (human GLUT4, UniProt P14672), which is highly expressed in adipose tissue, skeletal muscle, and cardiac muscle. Cloned in 1989 [2,3], GLUT4 is known for its dynamic intracellular recycling. Unlike GLUT1, which is constitutively localized at the plasma membrane to maintain basal glucose uptake, over 99% of GLUT4 is sequestered into highly specific intracellular clusters during fasting or resting conditions [4].

Both insulin and muscle contraction independently trigger the massive translocation of these storage vesicles to the plasma membrane, amplifying the cellular glucose uptake capacity by 20- to 40-fold [5]. This dynamic reserve system enables a rapid metabolic response to physiological requirements. Impairment of this cycle leads to severe pathologies, especially insulin resistance and T2DM, where compromised proximal insulin signaling inhibits GLUT4 recruitment, causing chronic hyperglycemia [6]. Fortunately, parallel, insulin-independent signaling networks, most potently triggered by physical exercise, can bypass these blockades to induce GLUT4 translocation [7]. Thus, exercise serves as a critical clinical intervention in T2DM. This review provides an updated synthesis of the structural principles governing GLUT4, the canonical and alternative pathways regulating its vesicular traffic, and the pathophysiological implications of tissue-specific trafficking defects.

## 2. Insulin Secretion by Pancreatic Beta Cells: Canonical Stimulation

**Figure 1:**
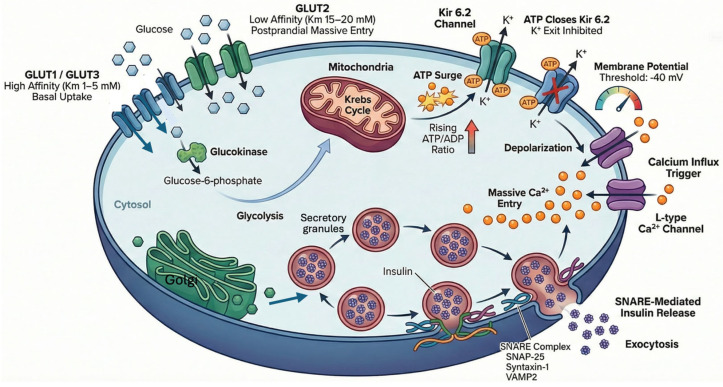
Schematic representation of synthesis and release of insulin in beta cells. Insulin is synthesized in the endoplasmic reticulum as preproinsulin and converted to proinsulin. In the Golgi apparatus, it is cleaved into mature insulin and C-peptide. It is then packaged into secretory granules, where it is stored until glucose stimulates its release into the bloodstream. A failure in this process contributes to type 2 diabetes.

Although this review focuses on peripheral GLUT4 regulation, a brief overview of glucose-stimulated insulin secretion (GSIS) by pancreatic beta cells will provide crucial context, as insulin is the primary endocrine trigger for canonical GLUT4 translocation (Figure 1). Postprandial extracellular glucose enters human beta cells primarily via the high-affinity facilitative transporters GLUT1 (SLC2A1) and GLUT3 (SLC2A3), with GLUT2 (SLC2A2) contributing during significant hyperglycemia [8,9,10,11]. Once in the cytosol, glucose is phosphorylated by glucokinase and undergoes rapid oxidative metabolism, significantly raising the intracellular ATP/ADP ratio. This energetic shift closes ATP-sensitive potassium channels (KATP), leading to membrane depolarization and the subsequent opening of voltage-dependent L-type calcium channels [12]. The resulting massive influx of cytosolic Ca^2+^ triggers the SNARE-mediated fusion (e.g., via SNAP-25, syntaxin-1, and VAMP2) of mature insulin secretory granules with the plasma membrane [13,14]. This tightly coupled stimulus-secretion mechanism ensures rapid insulin release into the portal circulation, providing the vital signal to activate the canonical PI3K/Akt pathway in peripheral insulin-sensitive tissues. Dysfunction in this secretory mechanism represents a primary defect in advanced type 2 diabetes.

## 3. Structure of GLUT4

**Figure 2:**
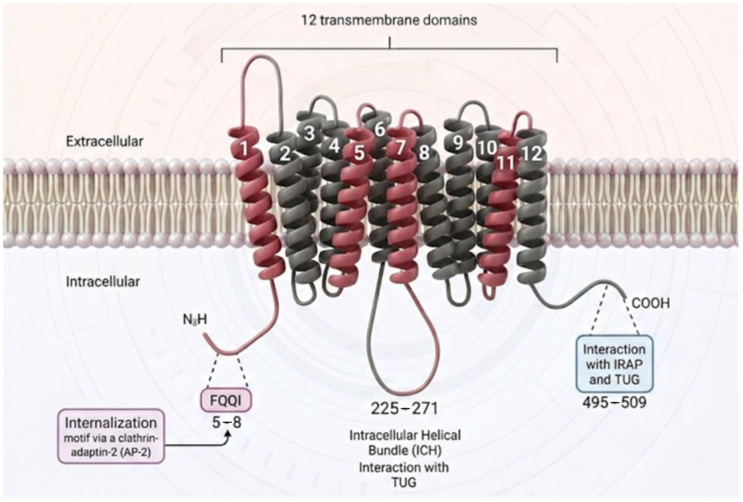
Schematic representation of the 12-transmembrane domain arrangement of the GLUT4 transporter in the lipid bilayer. Transmembrane helices 1, 5, 7, and 11 (highlighted in red) form the central substrate translocation pathway that constitutes the glucose channel. The main cytosolic segments involved in its post-translational regulation and vesicular trafficking are indicated, including the N-terminal motif associated with internalization, the intracellular helical domain, and the C-terminal region that interacts with retention and recycling proteins.

Human GLUT4 (Figure 2) is a 509-amino-acid facilitative transporter belonging to the SLC2A superfamily [15]. Through advances in cryo-electron microscopy (cryo-EM), artificial intelligence modeling (AlphaFold), and high sequence homology (>65%) with GLUT1 and GLUT3, the atomic structure of GLUT4 has been modeled with exceptional accuracy (0.95 Å and a 95% confidence level) [16,17]. It adopts a classic major facilitator superfamily fold: a barrel conformation composed of 12 transmembrane domains (TMDs), organized into two symmetric six-helix bundles connected by a large intracellular loop [18]. Both the N- and C-termini project into the cytosol, with the C-terminus harboring critical interaction platforms for vesicular trafficking proteins [19].

### 3.1. Kinetics and Rocking-Bundle Mechanism

**Figure 3:**
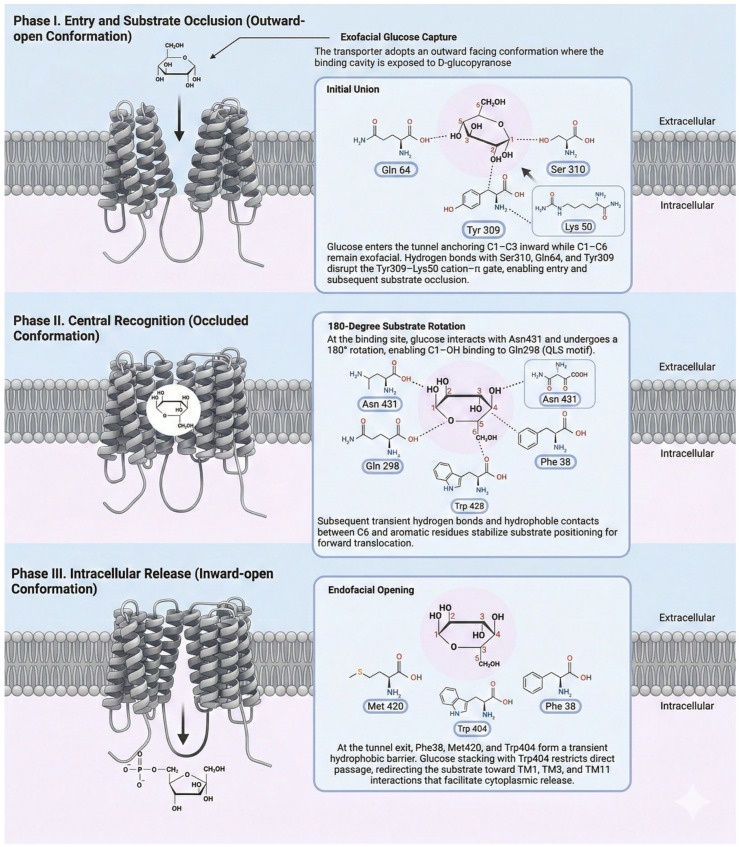
GLUT4-mediated glucose transport mechanism. Schematic representation of the three conformational phases of the glucose transport cycle, a simplified consensus model. In phase I (outward-open conformation), the exofacial cavity facilitates the initial capture of D-glucopyranose via polar interactions that promote its entry and partially occlude the substrate. In phase II (occluded state), the transporter adopts a closed conformation on both sides of the membrane, stabilizing glucose at the central binding site through a transient network of hydrophilic interactions and aromatic contacts that orient the substrate for translocation. In phase III (inward-open conformation), the endofacial opening reduces substrate affinity and favors its release into the cytosol.

The Km of GLUT4 (5.4 mM) is very close to the fasting blood glucose level (~5 mM), and the Vmax is 3.7 µm/mg/min-1 [20]. Insulin, by increasing the concentration of GLUT4 at the membrane surface, increases the Vmax for glucose entry or exit by up to 10 times, but not its Km [21]. The glucose influx and efflux system is kinetically symmetrical, with equal Km and Vmax [21]. Glucose transport follows the rocking-bundle mechanism (Figure 3). Essentially, the two 6-helix domains function as a rocking gate: they oscillate coordinately between three main conformational states: 1. Outward-open: The binding cavity is exposed to the extracellular space, allowing glucose entry. 2. Occluded: After substrate binding, the pore closes on both sides of the membrane, trapping the glucose. This ensures that the transported molecule is not released prematurely, thereby preventing a futile leak of ions or substrates. 3. Inward-open: The pore opens towards the cytosol, releasing glucose [16]. This cycle is reversible and depends solely on the glucose concentration gradient across the membrane [15].

### 3.2. Key Residues in the Binding Pore and Structural Relevance

The central hydrophilic pore is formed by conserved residues across TMs 1, 4, 5, 7, 10, and 11 and approximately 10–12 Å from the membrane surface [20]. Cryo-EM studies mapping the binding of D-glucopyranose reveal a highly specific hydrogen-bonding network involving residues such as Asn176, Gln298, Ser310, and Trp404 [20,22,23]. Understanding the precise architecture of this occluded state and the specific coordination of glucose provides the fundamental structural blueprint for rational drug design. Detailed mapping of this pore enables the development of isoform-specific competitive inhibitors (such as cytochalasin B analogs) and allosteric modulators that can selectively target GLUT4 in metabolic diseases and, potentially, in cancer biology.

The pore of GLUT4 provides a partially polar environment that stabilizes glucose during transport. The oxygen atoms of glucose’s hydroxyl groups form specific hydrogen bonds with Gln64, Gln177, Asn176, Gln298, Gln299, and Ser310 residues [22]. As glucose progresses, the hydrocarbon ring undergoes hydrophobic stacking interactions, notably with Trp404 and Met420 [22,23]. This coordinated network of transient polar and hydrophobic interactions strategically reduces the energy barrier for glucose translocation, facilitating its passive diffusion across the lipid bilayer [22]. For substrate release into the cytosol in the inward-open conformation, characterized by rearrangement of TM5 and TM11, the side chain of Lys^200^ on the endofacial side of TM5 projects into the binding pocket to electrostatically and sterically perturb the hydrogen-bonding network, lowering glucose affinity and promoting its release into the cytosol [16].

### 3.3. Post-Translational Modifications: Glycosylation and Ubiquitination

Beyond its primary sequence and localization motifs, post-translational modifications (PTMs) critically regulate GLUT4. N-linked glycosylation at Asn45 (in human GLUT4, corresponding to Asn57 in rodents) within the first exofacial loop is essential for proper protein folding, stability, and efficient sorting into the insulin-responsive GSV compartment [15]. This glycan chain prevents newly synthesized GLUT4 from undergoing premature proteasomal degradation, thereby ensuring an adequate half-life of the transporter pool [15]. Furthermore, GLUT4 is subject to specific ubiquitination at cytosolic lysine residues. This ubiquitination acts as a critical sorting signal rather than a mere degradation tag, facilitating interaction with GGA (Golgi-localized, γ-ear-containing, ARF-binding) adaptor proteins, which direct the sorting of GLUT4 from the trans-Golgi network and endosomal system into the specialized insulin-sensitive GSV compartment [17,18].

### 3.4. GSV Storage Sites and Cytoskeletal Anchoring

**Figure 4:**
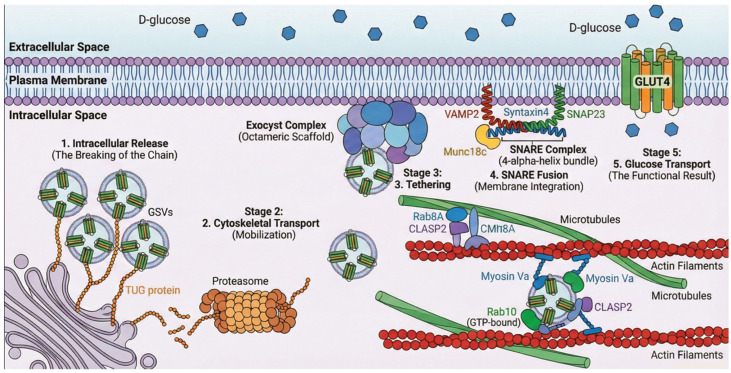
Representative scheme of GSV retention and release. The 5-stage journey of GLUT4. Note: This diagram illustrates a simplified linear progression; in vivo, these trafficking stages exhibit high redundancy and context-dependent regulatory crosstalk. Created in BioRender. Ramos-Jiménez, A. (2026) https://BioRender.com.

GLUT4 proteins are stored within specialized organelles called GLUT4 Storage Vesicles (GSVs) or Insulin-Responsive Vesicles (IRVs), which form a preformed pool designed for rapid mobilization [24,25]. In unstimulated cells, GSVs are retained near the Golgi apparatus and endoplasmic reticulum (Figure 4). Their journey to the surface involves: (1) Release from retention: under basal conditions, GSVs are anchored via interactions with proteins such as TUG (encoded by *ASPSCR1*). Insulin triggers the dissociation or endoproteolytic cleavage of TUG, releasing the vesicles [25]. (2) Transport: Motor proteins (e.g., KIF5B, Myosin Va) move released vesicles along the cytoskeleton [25]. (3) Docking and fusion: At the membrane, tethering is mediated by t-SNARE proteins (Syntaxin 4 and SNAP23), which bind the GSV’s v-SNARE (VAMP2) to complete membrane fusion [26,27].

## 4. Factors That Activate GLUT4 Translocation, Endocytosis, and Exocytosis

The translocation of GLUT4 occurs through several finely coupled mechanisms, both insulin-dependent and insulin-independent. These mechanisms activate a set of proteins involved in a signaling and phosphorylation cascade. These proteins intersect with two key substrates: AMP-activated protein kinase (AMPK) and Akt substrate of 160 kDa (AS160, encoded by the *TBC1D4* gene), which will be described below.

### 4.1. Intracellular Localization and Retention Motifs

A key feature of GLUT4 is its active recycling, regulated by specific sequence motifs (Figure 4). The N-terminal F^5^QQI^8^ motif (Phe^5^-Gln^6^-Gln^7^-Ile^8^) is an important trafficking signal [28,29]. It plays a dual role by interacting with the μ subunits of different adaptor protein complexes: it binds μ2 of the AP-2 complex to facilitate clathrin-dependent endocytosis [28,30] and it binds μ1 of the AP-1 complex for post-endosomal sorting [29]. This motif acts as a suboptimal internalization signal, combined with a switch in endocytic pathways, to help regulate GLUT4 surface levels in response to insulin [31]. A separate di-leucine motif (Leu^489^–Leu^490^) at the C-terminal domain is involved in intracellular retention and sorting from compartments such as the trans-Golgi network (TGN) [31]. This interaction recruits the clathrin-mediated endocytosis machinery, causing constitutive GLUT4 internalization. This process is the primary mechanism for surface removal, with a membrane half-life of about 5–10 min in the absence of insulin. The nearby C-terminal region (residues 495–509) contains critical signaling sites that control membrane retention in response to insulin and subsequent intracellular recycling. It interacts with proteins in GSVs, such as Insulin-Regulated Aminopeptidase (IRAP) and the structural anchoring protein TUG. The interaction with TUG is essential for keeping GSVs in a specialized compartment, possibly anchoring them to the cytoskeleton [19]. This domain also interacts with the exocytosis machinery, including SNARE proteins such as Syntaxin 4 and SNAP23, enabling specific fusion upon signaling [24].

### 4.2. Canonical Pathway of the Insulin-Stimulated Activation Cascade (PI3K/Akt/TBC1D4)

Insulin triggers a rapid, well-characterized signaling cascade to mobilize GSVs [32,33]. Upon binding its tyrosine kinase receptor (IR), the receptor autophosphorylates and recruits Insulin Receptor Substrate (IRS) proteins [34]. Phosphorylated IRS-1 recruits class Ia PI3K, generating the lipid second messenger PIP_3_ [35]. PIP_3_ recruits PDK1 and Akt (PKB) to the membrane. Akt is fully activated via phosphorylation at Thr308 by PDK1 and at Ser473 by mTORC2 [36]. Cellular glucose uptake is predominantly in skeletal muscle (~80%), with adipose and cardiac muscle contributing less [32,33]. Insulin binding to the α subunit of its tyrosine kinase receptor causes a conformational change in the β subunit, exposing activation and autophosphorylation sites on tyrosine residues. This triggers a phosphorylation cascade culminating in the activation of the protein kinase Akt, also known as PKB. Akt then phosphorylates effector proteins, such as AS160, inactivating their GAP (GTPase-activating protein) activity towards small Rab proteins. Activated (GTP-bound) Rab10 and Rab14 then recruit GSVs to the plasma membrane [37]. The fusion site with the membrane involves specific interactions between SNARE proteins on the vesicle (such as VAMP2) and on the plasma membrane (such as Syntaxin4 and SNAP23), facilitating transporter exposure on the cell surface [38].

**Figure 5:**
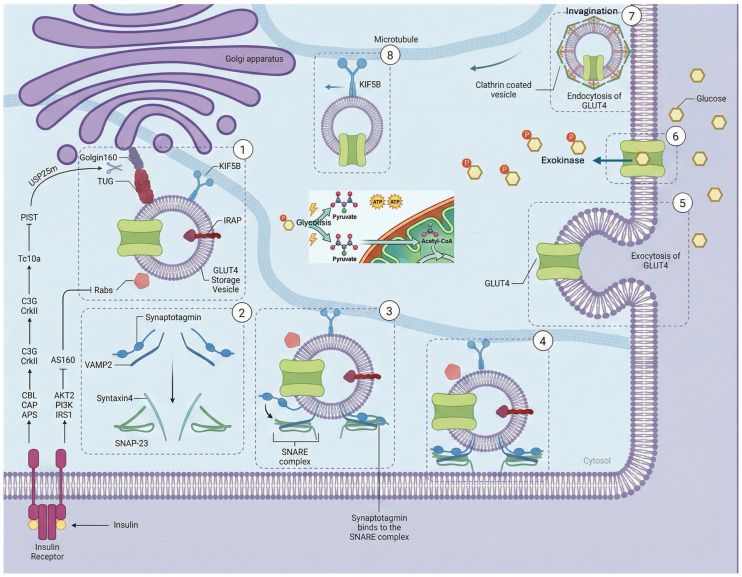
Representative model of the coordinated events that regulate GLUT4 mobilization and recycling. (1) Release of storage vesicles (GSVs) from their intracellular retention state through activation of Rab proteins and dissociation of anchoring complexes; (2) organization of the fusion machinery at the plasma membrane, including t-SNARE proteins; (3) vesicular approach and initial assembly of the SNARE complex with VAMP2 involvement; (4) stabilization of docking in the cortical region; (5) vesicular fusion and insertion of GLUT4 into the plasma membrane; (6) facilitated glucose transport; (7) internalization of the transporter via clathrin-dependent endocytosis; and (8) cytoskeleton-associated intracellular recycling and redirection for subsequent reuse.

IRS proteins (1–4) are recruited to the phosphorylated domains of the IR via their PTB (phosphotyrosine-binding) domains and are phosphorylated on multiple tyrosine residues. Phosphorylation of Tyr612 and Tyr632 residues on IRS-1 recruits and activates class Ia PI3K (regulated by the p85 subunit, catalyzed by p110) [39]. Activated PI3K phosphorylates phosphatidylinositol (4,5)-bisphosphate (PIP_2_) at the 3-position of the inositol ring, generating phosphatidylinositol (3,4,5)-trisphosphate (PIP_3_). PIP_3_ acts as a lipid second messenger that recruits proteins with pleckstrin-homology (PH) domains to the plasma membrane [35]. Both PDK1 (3-phosphoinositide-dependent protein kinase-1) and protein kinase B (Akt/PKB) possess PH domains that bind PIP_3_ with high affinity, recruiting them to the plasma membrane. PDK1 phosphorylates Akt at the key residue Thr308 in the activation loop. This phosphorylation is necessary but not sufficient for maximum activity (Figure 5). A second phosphorylation, typically on Ser473 (in the C-terminal hydrophobic domain), is mediated by the mTORC2 complex (mammalian target of rapamycin complex 2) and is essential for full activation and substrate specificity of Akt [36].

### 4.3. TBC1D4 (AS160) as a Basal Translocation Suppressor

The most relevant Akt substrate for GLUT4 translocation is TBC1D4. In skeletal muscle, its close relative, TBC1D1, plays a parallel and often predominant role [40]. TBC1D4 is a GTPase-activating protein (GAP). Its GAP domain specifically targets a subset of small Rab GTPases, including Rab8A, Rab10, Rab13, and Rab14. Under basal conditions, the GAP activity of TBC1D4 catalyzes the hydrolysis of GTP bound to these Rabs to GDP, keeping them inactive (GDP-bound). Since GTP-bound Rabs are master regulators that recruit effectors for vesicle movement, anchoring, and fusion, this GAP activity constitutes a molecular brake that keeps GSVs immobilized and sequestered [41]. Akt phosphorylates TBC1D4 on at least 6 residues (Ser318, Ser341, Ser570, Ser588, Thr642, Ser751, in humans). Phosphorylation at Ser588 and Thr642 appears to be particularly critical [42]. The phosphorylation of Ser588 and Thr642 induces a conformational change that inhibits TBC1D4’s GAP activity, allowing Rabs to transition to their active, GTP-bound state. Concurrently, the physical tether TUG is cleaved, releasing the vesicles from the Golgi matrix [19]. Active Rabs then recruit motor complexes to navigate the actin cytoskeleton, and the exocyst complex physically tethers the vesicle to the plasma membrane, preparing it for SNARE-mediated fusion via VAMP2, Syntaxin 4, and SNAP23 [43,44,45,46,47].

The fusion of GSVs to the membrane, mediated by Soluble NSF Attachment Protein Receptors (SNARE), is the final and irreversible step. SNAREs located on GSVs (v-SNAREs) and the target membrane (t-SNAREs) form a stable 4-alpha-helix bundle. The formation of the trans-SNARE complex (VAMP2-Syntaxin4-SNAP23) between the two membranes exerts a mechanical force that overcomes the repulsion between lipid bilayers, fusing them. The regulatory SM protein Munc18c is essential: it acts as a scaffold and chaperone, facilitating the correct folding of Syntaxin 4 and its interaction with VAMP2 [47]. After fusion, GLUT4 is incorporated into the plasma membrane; its transport pore is exposed to the extracellular space, allowing it to begin transporting glucose. This process is highly efficient and recruits most of the intracellular GLUT4 pool within 10–30 min.

## 5. Alternative and Contraction-Mediated Pathways (AMPK-Dependent and -Independent)

**Figure 6:**
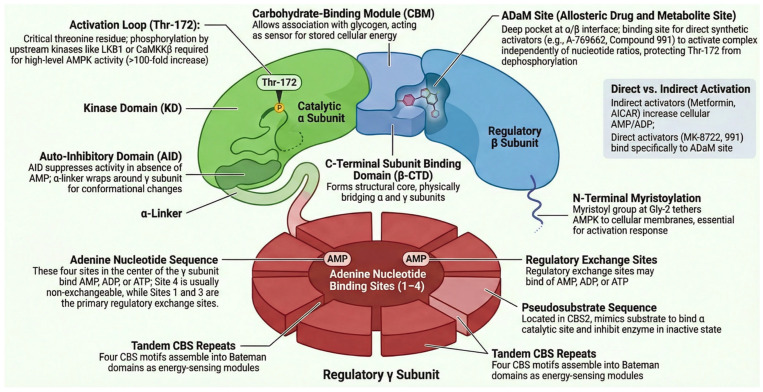
Schematic representation of the human AMPK complex. The magnification shows the detail of the α2 subunit activation loop, Thr172 in its phosphorylated state (pThr172), with the phosphate group coordinated by hydrogen bonds that stabilize the activation segment. This post-translational modification is essential for the kinase domain to adopt the catalytically competent conformation. Created in BioRender. Ramos-Jiménez, A. (2026) https://BioRender.com.

AMPK (Figure 6) exists as a heterotrimer composed of a catalytic α subunit, a scaffolding β subunit, and a regulatory γ subunit, with multiple isoforms of each (α1, α2; β1, β2; γ1, γ2, γ3), conferring tissue and functional diversity. The α subunit contains the N-terminal kinase domain, an autoinhibitory domain (AID) that keeps the enzyme inactive under basal conditions, and a binding domain for the β/γ subunits at its C-terminal end. The β subunit acts as a central scaffold, containing a carbohydrate-binding module (CBM) that directs AMPK to glycogen granules, localizing it near its substrate and energy sources [48]. The γ subunit is the energy-sensing module. It contains four CBS (cystathionine β-synthase) motifs that form three binding sites (sites 1, 3, and 4) for adenine nucleotides. The competitive binding of these ligands constitutes the core of canonical regulation [49]. However, the central activation event of AMPK is the phosphorylation of the Thr172 residue in the activation loop of the α subunit. The degree of phosphorylation at Thr172 directly determines catalytic activity and is governed by a dynamic balance between upstream kinases and phosphatases.

While the Akt/TBC1D4 pathway is the canonical route for insulin, physical exercise acts as a potent physiological stimulus for glucose uptake, functioning entirely independently of insulin [50]. Knockout mice lacking the AMPK catalytic subunits maintain normal insulin-stimulated glucose uptake, demonstrating that the pathways are mechanistically distinct [51,52]. During contraction, the muscle relies on redundant cascades converging on the TBC1D1/TBC1D4 RabGAPs. Therefore, it is crucial to highlight the existence of pathways completely independent of AMPK for GLUT4 translocation and glucose uptake. This demonstrates that AMPK is not a necessary intermediary in insulin signaling (Figure 6).

### 5.1. Muscle Contraction and Energy Stress Pathway

The glucose uptake in skeletal muscle induced by exercise is complex and redundant, converging on the same endpoints as insulin, AMPK, and TBC1D1. During exercise, muscle contraction engages overlapping signaling axes that bypass the insulin receptor entirely:

- AMPK-Dependent Mechanisms: AMPK is the master sensor of cellular energy status [53]. An increasing AMP/ATP ratio allosterically activates AMPK and promotes its phosphorylation at Thr172 by upstream kinases like LKB1 [48,49]. Acutely, AMPK directly phosphorylates TBC1D1 at specific residues (e.g., Ser237 in humans, corresponding to Ser231 in mice), suppressing its GAP activity and facilitating Rab activation [54,55,56].
- AMPK-Independent Mechanisms (CaMKII and p38 MAPK): Genetically modified mice (AMPKα1/α2 dKO) demonstrate that AMPK accounts for roughly 40–50% of contraction-induced glucose uptake, proving that it is a parallel contributor rather than the sole intermediary of exercise signaling [51]. Depolarization of T-tubules releases a flood of sarcoplasmic Ca^2+^, activating CaMKII. Evidence, primarily from experimental models, indicates that CaMKII largely acts through a parallel Rac1-GEF (Kalirin)/Akt signaling axis to modulate the actin cytoskeleton for vesicular transport [57]. Concurrently, mechanical stress and reactive oxygen species (ROS) selectively activate the p38γ (encoded by *MAPK12*) and p38δ (encoded by *MAPK13*) isoforms [58,59,60]. These kinases phosphorylate TBC1D1 and TBC1D4 at exercise-specific residues distinct from those targeted by Akt [61]. Human phosphoproteomic studies confirm that exercise induces a unique phosphorylation signature on TBC1D1, mediated by a synergy between the AMPK and p38 networks [62,63].

### 5.2. Upstream Regulatory Kinases

Upstream phosphorylation of AMPKα at Thr172 is absolutely essential for full activation (more than 100-fold) [49]. Its phosphorylation is carried out by at least three main kinases, whose relevance depends on cell type and stimulus: (1) Liver Kinase B1 (LKB1/STK11): This is the most important constitutive kinase for the response to energy stress. LKB1 forms a stable, obligatory complex with the accessory proteins STRAD and MO25, which are essential for its activity and localization [52]. LKB1 is constitutively active and monitors cellular energy status, phosphorylating AMPK when AMP/ADP binding favors an active conformation. Metformin, which mildly inhibits mitochondrial respiratory chain complex I, subtly increases the AMP:ATP ratio and activates AMPK in an LKB1-dependent manner [64]. Fasting, caloric restriction, and exercise contribute significantly to AMPK activation during contraction, especially in response to ATP consumption. (2) During muscle contraction, the increase in intracellular calcium ([Ca^2+^]i), independently of changes in adenine nucleotides, activates Calcium/Calmodulin-dependent Protein Kinase Kinase β (CaMKKβ), which in turn activates AMPK. This pathway is particularly relevant in neurons, where depolarization and Ca^2+^ entry activate AMPK to meet the energy demand of synaptic signaling. During muscle contraction, it works in synergy with LKB1. While LKB1 responds to ATP deficit, CaMKKβ responds to the initial Ca^2+^ signal released during contraction. Studies in mice with muscle-specific LKB1 deletion show that approximately 60% of contraction-induced AMPK activation is preserved, mainly due to CaMKKβ [55].

### 5.3. Role of AMPK in GLUT4 Regulation: Contextual Contribution

As mentioned above, AMPK is not the main pathway for insulin- or contraction-mediated activation but rather a modulatory contributor that augments other signals. Acutely, AMPK activates direct phosphorylation of TBC1D1 at residue Ser237 (Ser231 in mouse) [65]. This phosphorylation inhibits TBC1D1 GAP activity, facilitating Rab activation and GLUT4 translocation. Its effect is additive to phosphorylation mediated by CaMKII and p38 MAPK during contraction. In muscle, the contribution of AMPK to contraction-induced glucose uptake is approximately 30–40%, as demonstrated by the partial reduction observed in AMPKα1/α2 dKO mice [51]. AMPK can chronically induce GLUT4 gene expression in the long term through indirect mechanisms, such as activation of PGC-1α, since AMPK phosphorylates and activates PGC-1α, the master coactivator of mitochondrial biogenesis and metabolic gene expression, including SLC2A4 [66], and epigenetic modulation: AMPK phosphorylates and inhibits histone deacetylases like HDAC5, alleviating chromatin repression on the GLUT4 promoter and other metabolic genes [67].

AMPK is also not simply an exercise mimetic. Its chronic and systemic activation can have adverse effects, such as: (1) Lack of tissue specificity, as classic activators (AICAR, metformin) act in multiple organs. (2) Undesired cardiac activation, since potent AMPK activation in the heart (e.g., with MK-8722) can induce cardiac hypertrophy and glycogen accumulation, effects not observed with exercise [68]. (3) Inhibition by counterregulatory hormones, such as glucagon and adrenaline, activates PKA, which phosphorylates AMPKα at Ser173 (adjacent to Thr172), inhibiting AMPKα activation by LKB1. This prevents AMPK from activating glucose uptake when the body needs to mobilize energy [69]. Therefore, targeting AMPK lies in developing complex-specific allosteric activators (e.g., acting only on complexes containing the muscle α1β1γ1 subunit) or safer indirect modulators. AMPK activation by exercise may be slightly attenuated in obesity and T2DM, possibly due to inflammation or endoplasmic reticulum stress. However, its activation through drugs like metformin remains effective and constitutes one basis of therapy [70].

## 6. Acute vs. Chronic Exercise: GLUT4 Translocation and Synthesis

**Figure 7:**
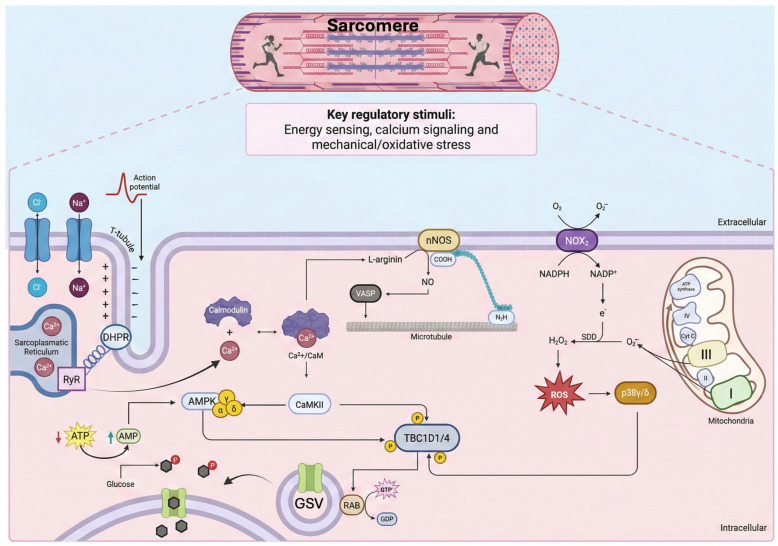
Convergence of exercise-induced signals on the TBC1D1/4–Rab–GLUT4 axis. Membrane depolarization [from negative (−) to positive (+)] and excitation-contraction coupling generate calcium signals, energy stress, and oxidative stress, thereby activating CaMKII, AMPK, and p38γ/δ. These kinases converge on the phosphorylation of TBC1D1/4, promoting the activation of Rab GTPases and the translocation of GLUT4 to the plasma membrane in an insulin-independent manner. Created in BioRender. Ramos-Jiménez, A. (2026) https://BioRender.com.

Both insulin and exercise have the same short-term stimulatory effect on GLUT4 synthesis, but exercise has a stronger long-term effect [49]. The GLUT4 translocation induced by insulin and muscle contraction occurs via distinct mechanisms (Figure 7): the former involves the insulin signaling cascade and is dependent on PI3K activation; the latter is independent of PI3K and involves AMPK activation.

While acute exercise mobilizes existing GSVs, chronic endurance exercise expands the absolute cellular GLUT4 reserve [71,72]. Muscle contraction activates AMPK and p38 MAPK, which converge on PGC-1α (the master coactivator of energy metabolic genes) [66]. PGC-1α lacks intrinsic DNA-binding capacity; instead, it interacts with the transcription factor MEF2 at the SLC2A4 promoter. PGC-1α recruits chromatin-remodeling complexes (SWI/SNF) and histone acetyltransferases (HATs), establishing a highly permissive chromatin state that can elevate GLUT4 mRNA transcription two- to three-fold [65].

### Exercise-Induced Metabolic Memory

Crucially, exercise induces lasting epigenetic modifications, a “metabolic memory” [73]. A single exercise session triggers rapid increases in activating histone marks (e.g., H3K4me3 and H3K27ac) at the SLC2A4 promoter [74]. Additionally, exercise-activated kinases promote the nuclear export of class II histone deacetylases (HDAC4/5), alleviating baseline transcriptional repression [56]. With chronic training, these epigenetic marks stabilize, sustainably elevating basal GLUT4 content and expanding the total GSV pool available for future stimuli, forming the molecular basis for the long-term insulin-sensitizing effects of regular exercise [75,76]. This functional memory not only explains the sustained improvements in insulin sensitivity and glucose homeostasis observed after training but also underscores the superiority of exercise as an intervention to counteract specific defects in insulin signaling in metabolic diseases, offering a resilient and potentially effective mechanism for glycemic control [7,75,76]. These epigenetic changes can persist for hours or even days after the acute stimulus ceases, priming the gene for more efficient activation. With chronic training, these marks can become more stable, contributing to the sustained phenotypic adaptation observed in many athletes.

## 7. Molecular Pathophysiology of T2DM

T2DM is not a generalized failure of glucose uptake but rather an imbalance in a specific regulatory pathway [37,77]. Skeletal muscle insulin resistance involves an early, selective alteration of the canonical PI3K/Akt signaling cascade. Intracellular lipids and cytokines activate kinases (PKCθ, JNK, IKKβ) that promote inhibitory serine phosphorylation of IRS-1. This prevents essential tyrosine phosphorylation and drives IRS-1 degradation [77,78,79,80]. Consequently, PI3K recruitment, PIP3 generation, and Akt activation are impaired [37,77]. Inhibited Akt fails to phosphorylate TBC1D4; the resulting excessive RabGAP activity keeps Rab GTPases inactive, creating a bottleneck that prevents massive GLUT4 translocation to the plasma membrane [37,81]. Coexisting inefficient vesicular trafficking [81] and reduced adipocyte GLUT4 mRNA [82] further diminishes membrane GLUT4 density, precipitating fasting and postprandial hyperglycemia [77,78]. Protectively, insulin-independent muscle contraction pathways (Ca^2+^/CaMKII and p38 MAPK γ/δ) remain functional [55,83], explaining why T2DM patients effectively increase glucose uptake via exercise.

### 7.1. Alterations in GSV Storage, Retention, and Fusion

Translocation of GLUT4, stored in intracellular GSVs under basal conditions, can be altered by defects in proteins of the TUG (Tether containing UBX domain for GLUT4) family or AS160 or in the GAPDH protein, compromising the formation, stability, or intracellular retention of GLUT4 clusters, and, as a consequence, GLUT4 is not efficiently retained and leaks into constitutive recycling pathways, reducing the pool specifically mobilizable by insulin. Yu et al. 2012 showed that the TUG protein mediates GLUT4 retention in a storage compartment distinct from the recycling endosome [19]. Disruption of TUG leads to increased basal translocation and decreased insulin response.

Even when GSVs are translocated to the cell cortex, they require the SNARE machinery (e.g., VAMP2, Syntaxin4, SNAP23) to fuse. Altered expression or function of these components, or their regulators (such as Munc18c), will prevent GLUT4 from fusing with the membrane. Consequently, GSVs accumulated in the periphery cannot release their contents, a phenomenon sometimes termed “docking without fusion”. The work of Kawanishi et al. in adipocytes from diabetic db/db models showed decreased expression of syntaxin 4 and SNAP23, correlated with impaired fusion of GLUT4-containing vesicles [84].

### 7.2. Alterations in Endocytosis and Recycling

Endocytosis and re-sorting of GLUT4 into GSVs are crucial steps in its recycling. Defects in endocytosis (e.g., due to alterations in clathrin, dynamin, or the internalization motif of GLUT4) can lead to loss of GLUT4 from the cell surface or trafficking to degradative compartments. This defect can cause both chronic loss of the transporter and an inability to replenish the GSV pool after stimulation. Blot and McGraw 2008 demonstrated that a single residue (Phe5) in the intracellular loop of GLUT4 is critical for its efficient internalization [28]. Mutations in this motif disrupt the entire cycle and insulin response [28]. Therefore, coordination in the cortical actin network is essential for the translocation and anchoring of GSVs near the plasma membrane. In states of insulin resistance, actin organization is altered, hindering final vesicle movement and fusion and contributing to the post-receptor defect. Omata et al. 2000 showed that insulin reorganizes actin in adipocytes and that pharmacological disruption of this cytoskeleton blocks GLUT4 translocation, independently of insulin signaling [85]. In summary, intrinsic defects in the GLUT4 cycle represent a fundamental cellular pathology that goes beyond mere insulin signaling and partly explains the irreversibility and progression of insulin resistance in key tissues.

## 8. Pharmacological Regulators

Knowledge of the mechanisms indicated above has driven the development of specific drugs that act as direct and indirect activators of GLUT4 translocation and glucose uptake. As direct activators (binding to the complex), we have AICAR, a prodrug that is intracellularly converted to ZMP, an AMP analog that activates AMPK in an LKB1-dependent manner, and A-769662 and 991, which bind to a distinct allosteric site at the α-β interface, stabilizing the phosphorylated form and inhibiting dephosphorylation. Their action can be independent of LKB1. As indirect activators (inducers of energy stress), we have metformin, a mild inhibitor of mitochondrial complex I. Its activation of AMPK is indirect, mediated by increased AMP/ADP and LKB1. Berberine and resveratrol are natural compounds with multiple mechanisms of action, including mitochondrial inhibition and modulation of SIRT1/AMPK. However, due to complex physiology, all drugs are non-specific and present various complications. For example, chronic and systemic AMPK activation can have adverse effects (e.g., cancer, autoimmune problems, cardiac hypertrophy) [86]. The future will lie in developing complex-specific activators (e.g., for muscle α1β1γ1) or tissue-targeted delivery systems.

### Agonists of the Nuclear Receptor PPARγ

Thiazolidinediones (TZDs: rosiglitazone, pioglitazone) improve insulin sensitivity independently of acute PI3K/Akt and AMPK signaling, acting at the level of gene regulation [83]. Ligand-bound PPARγ heterodimerizes with RXR on *SLC2A4* promoters, recruiting coactivators like PGC-1α to induce a massive increase in adipocyte GLUT4 transcription. Conversely, in T2DM, chronic hyperinsulinemia (via mTOR/S6K1-mediated mRNA instability) and macrophage-secreted cytokines (activating JNK and IKKβ/NF-κB) directly repress *SLC2A4* expression [70]. Furthermore, diminished Akt signaling retains unphosphorylated FOXO1 in the nucleus, repressing *SLC2A4* while activating hepatic gluconeogenic genes [86,87], exacerbated by glucotoxic histone modifications.

Highlighting novel regulatory paradigms, AMPK modulation extends beyond canonical adenylate charge. Contextually, stress-activated TAK1 [55,64] and reactive oxygen species, specifically via oxidation of the human α-subunit Cys299/Cys304, activate AMPK. Crucially, catabolic hormones enforce vital negative feedback: PKA phosphorylates AMPKα at Ser173, sterically preventing LKB1-driven Thr172 activation [69].

Furthermore, a newly clarified mechanism redefines the AMPK-SIRT1 energy-redox loop [66,88]. Rather than SIRT1 directly activating AMPK, AMPK acts as the primary upstream sensor. AMPK stimulates NAMPT expression [89], driving an NAD+ surge that activates SIRT1 [90]. SIRT1 subsequently deacetylates targets like PGC-1α and FOXO, promoting mitochondrial biogenesis and stress resistance [88,90].

## 9. Integrative Synthesis: Pathway Convergence and Tissue-Specific Divergence

The GLUT4 regulatory system does not operate via separate compartments. A critical synthesis reveals essential differences in GLUT4 regulation across tissues and physiological states:

- Tissue Specificity: Adipocytes rely almost exclusively on the canonical insulin-PI3K-Akt axis and utilize Rab10 for GLUT4 translocation. In contrast, skeletal muscle integrates both insulin and contraction-mediated signals, utilizing a broader array of upstream kinases (AMPK, CaMKII, p38 MAPK) and distal effectors (*TBC1D1* and *TBC1D4*, which engage Rab8A, Rab13, and Rab14) [46].
- Acute vs. Chronic Stimuli: Acute exercise relies on transient phosphorylation events (e.g., *TBC1D1/D4* inhibition) to rapidly mobilize pre-existing GSVs. Conversely, chronic endurance training induces sustained epigenetic and transcriptional adaptations that expand the total cellular expression of GLUT4, permanently elevating metabolic capacity.
- Pathway Synergy: Exercise and insulin pathways frequently overlap. A single 30 min bout of exercise establishes a unique phosphosignature on RabGAPs that “primes” the muscle, making it hypersensitive to subsequent insulin stimulation. This synergy explains the robust 24–48 h window of post-exercise insulin sensitivity [61,63,91,92,93,94,95,96].
- Disease Disconnects: In the pathophysiology of T2DM, a fundamental topological disconnect occurs. Proximal insulin signaling (IRS-1/Akt) is severely blunted by lipotoxicity, but the distal vesicular trafficking machinery and alternative contraction-activated pathways remain largely viable. Although some advanced diabetic models exhibit intrinsic structural defects [84], this selective signaling impairment explains why physical activity remains highly effective even in advanced states of insulin resistance.

In summary, when acute exercise becomes chronic, it increases *SLC2A4* gene expression, which encodes GLUT4, and optimizes the actions of several pathways and proteins implicated in energy metabolism, such as the Krebs cycle, respiratory chain, PGC-1α (a master regulator of mitochondrial and metabolic biogenesis), MEF2, and HDAC. It improves the biogenesis and trafficking machinery of GSV on the cytoskeleton. Chronic exercise also increases the expression of key cycle proteins, such as TUG (for retention), SNAREs (for fusion), and cytoskeletal components, thereby optimizing the entire system. By stimulating vesicular machinery, exercise favors a more efficient cycle of endocytosis, sorting, and re-storage of GLUT4, thereby avoiding its degradation or loss through constitutive pathways and reversing insulin resistance.

## 10. Therapeutic Perspectives and Future Research Directions

The biochemical study of canonical and alternative pathways regulating GLUT4 and glucose uptake will help us understand the complex molecular mechanisms underlying insulin resistance and type 2 diabetes. Future treatments must leverage this knowledge to identify how the type and form of short- or long-term exercise could be effective in reducing weight and decreasing these diseases. Similarly, it is necessary to find alternative, affordable ways to allow people with limited mobility or who are bedridden to exercise and have muscle contractions with electrical stimulation. With this decrease comes the metabolic deterioration of type 2 diabetes.

### Development Proposals

- Specific p38 MAPK γ/δ Activators: Given the fundamental role of p38γ/δ in exercise-induced GLUT4 translocation and its preservation in T2DM, developing small-molecule allosteric activators targeting these specific isoforms could bypass inflammatory risks associated with ubiquitous p38α/β activation.
- Complex-Specific AMPK Modulators: Because pan-AMPK activators (like MK-8722) can induce unwanted cardiac hypertrophy, precision drugs must selectively target the α1β1γ1 complex predominant in skeletal muscle (e.g., PF-06409577) [84,87]. Alternatively, tissue-activated prodrugs cleaved uniquely by muscle enzymes could restrict drug activity to myocytes.
- Distal Trafficking Modulators: Identifying molecules that stabilize the GTP-bound active conformation of Rab8A and Rab10—effectively bypassing both Akt and TBC1D4/1—offers a direct route to forcibly untether GSVs regardless of proximal resistance.
- Epigenetic Editing: Advanced strategies, such as CRISPR/dCas9 systems coupled with activating domains (e.g., p300) or targeted HDAC5 inhibitors, could permanently rewrite the repressive chromatin landscape at the *SLC2A4* promoter in diabetic patients, mimicking the persistent metabolic memory of chronic athletic training without requiring continuous physical stimulus [97].

To improve or induce sustained adaptations, advanced epigenetic therapies offer revolutionary potential. For example, GLUT4 overexpression via viral vectors: preclinical studies with adeno-associated (AAV) vectors expressing GLUT4 under muscle-specific promoters (e.g., myosin light chain promoter) have demonstrated improved glucose tolerance and insulin sensitivity in diabetic mouse models [98]. Challenges include immunogenicity, efficient delivery to sufficient muscle mass, and long-term control of expression. Epigenetic Editing and Modulation: to imprint a metabolic memory similar to exercise without the need for continuous exercise. Researchers could develop targeted transcriptional activators, such as CRISPR/dCas9 systems coupled to transcription activation domains (e.g., dCas9-VPR) or enzymes that deposit histone activation marks (e.g., dCas9-p300), to specifically target the SLC2A4 gene promoter and increase its expression in a durable, regulable manner [97]. For example, researchers could develop drugs that inhibit specific histone deacetylases (HDACs). Since AMPK phosphorylates and inhibits HDAC5, selective HDAC5 inhibitors could be developed to alleviate chromatin repression of *SLC2A4* and other metabolic genes, thereby replicating this effect.

## 11. Conclusions

The glucose transporter GLUT4 serves as a central node in metabolic physiology, whose complex regulation underscores its critical importance for energy homeostasis. This review integrated current evidence, revealing an extraordinary system of sophistication and redundancy, synthesized in the following 6 points.

1. Architecture and Function: GLUT4 is an exquisitely designed molecular machine whose alternating access transport mechanism is finely tuned. Its localization motifs (F^5^QQI^8^, N-terminal domain) determine its dynamic trafficking between a specialized intracellular storage compartment (GSVs) and the plasma membrane.
2. Canonical Insulin Pathway: The PI3K/Akt/TBC1D4 axis represents the high-fidelity system for acute translocation in the fed state. Its activation triggers an orchestrated cascade that releases the molecular brake imposed by the GAPs TBC1D4/D1, allowing Rab GTPases to orchestrate the transport, anchoring, and fusion of GSVs.
3. Insulin-Independent Pathways: Muscle contraction activates parallel, robust mechanisms, driven by Ca^2+^ signals (via CaMKII) and mechanical/metabolic stress (via p38 MAPK γ/δ). These pathways converge on the inhibition of the GAPs TBC1D1/D4 via distinct phosphorylation targets, ensuring glucose uptake in response to acute energy demand, independent of hormonal status.
4. AMPK as an Integrative Sensor: AMPK acts as a guardian of energy status, activated by both ATP deficit (via LKB1) and calcium (via CaMKKβ). It contributes to GLUT4 translocation and its long-term expression but is not essential for the main insulin or contraction pathways.
5. Transcriptional Regulation: The expression of the *SLC2A4* gene is controlled by long-term programs, with the exercise-induced PGC-1α/MEF2 axis being the most potent physiological regulator and the nuclear receptor PPARγ being the direct pharmacological target
6. Given the significant positive effects of physical exercise and its synergy with drugs to reduce insulin resistance, it is necessary to require that every health institution have appropriate facilities and equipment so that every patient with metabolic diseases receives, in addition to pharmacological treatment, supervised physical exercise.

## Data Availability

The data used in this study can be accessed at https://www.dropbox.com/scl/fo/dmscsms3qm3644b9kfddp/AOhWk9qTQOEv3Sa5aj3w42M?rlkey=5udz5jckei5pdw2rmy4rp26sp&dl=0 (accessed on 10 February 2026). Biorender agreement numbers: *UX29L8462D*, *BJ29L84DEI*, *ZA29L84JIW*, *XK29L83XP7*.

## Abbreviations

The following abbreviations are used in this manuscript:

AAV	Adeno-associated vector
ADP	Adenosine diphosphate
AICAR	5-Aminoimidazole-4-carboxamide ribonucleotide
AMP	Adenosine monophosphate
AMPK	AMP-activated protein kinase
AP-1/AP-2	Adaptor protein complexes 1 and 2
AS160	Akt substrate of 160 kDa (also known as TBC1D4)
ATP	Adenosine triphosphate
CaM	Calmodulin
CaMK/CaMKII	Calcium/calmodulin-dependent protein kinase II
CaMKKβ	Calcium/calmodulin-dependent protein kinase β
CBM	Carbohydrate-binding module
CBS	Cystathionine β-synthase
CCB	Cytochalasin B
CRISPR/dCas9	Clustered Regularly Interspaced Short Palindromic Repeats/dead Cas9
DHPR	Dihydropyridine receptor
dKO	Double knockout
EPAC	Exchange protein directly activated by cAMP
GAP	GTPase-activating protein
GLP-1	Glucagon-like peptide-1
GLUT1/GLUT2/GLUT3/GLUT4	Glucose transporter types 1, 2, 3, and 4
GSIS	Glucose-stimulated insulin secretion
GSV	GLUT4 storage vesicle
HATs	Histone acetyltransferases
HDAC/HDAC4/HDAC5	Histone deacetylase
IKKβ	Inhibitor of nuclear factor-kappa B kinase subunit beta
IR	Insulin receptor
IRAP	Insulin-regulated aminopeptidase
IRS/IRS-1	Insulin receptor substrate (1-4)
IRV	Insulin-responsive vesicle
JNK	c-Jun N-terminal kinase
KATP	ATP-sensitive potassium channel
LKB1	Liver Kinase B1
MAPK	Mitogen-activated protein kinase (specifically p38 MAPK)
MEF2/MEF2A	Myocyte enhancer factor 2/2A
MK2	MAPK-activated protein kinase 2
mTORC2	Mammalian target of rapamycin complex 2
NAD+/NADH	Nicotinamide adenine dinucleotide (oxidized and reduced forms)
NAMPT	Nicotinamide phosphoribosyltransferase
nNOS	Neuronal nitric oxide synthase
PDK1	3-Phosphoinositide-dependent protein kinase-1
PGC-1α	Peroxisome proliferator-activated receptor gamma coactivator 1-alpha
PH domain	Pleckstrin-homology domain
PI3K	Phosphatidylinositol 3-kinase
PIP2	Phosphatidylinositol (4,5)-bisphosphate
PIP3	Phosphatidylinositol (3,4,5)-trisphosphate
PKA	Protein kinase A
PKB	Protein kinase B (also known as Akt)
PKCθ	Protein kinase C theta
PPARγ	Peroxisome proliferator-activated receptor gamma
PPRE	PPAR response element
PTB	Phosphotyrosine-binding
ROS	Reactive oxygen species
RXR	Retinoid X receptor
RyR	Ryanodine receptor
SIRT1	Sirtuin 1
SLC2A/SLC2A1-4	Solute carrier family 2 (facilitative glucose transporter) members
SNAP23/SNAP-25	Synaptosome-associated proteins 23 and 25
SNARE	Soluble NSF Attachment Protein Receptor
T2DM	Type 2 diabetes mellitus
TBC1D1/TBC1D4	TBC1 domain family members 1 and 4
TGN	Trans-Golgi network
TM/TMD	Transmembrane/Transmembrane domain
TNF-α	Tumor necrosis factor alpha
TUG	Tether containing UBX domain for GLUT4
VAMP2	Vesicle-associated membrane protein 2
VASP	Vasodilator-stimulated phosphoprotein
